# Inhibition of Pro-Fibrotic Molecules Expression in Idiopathic Pulmonary Fibrosis—Derived Lung Fibroblasts by Lactose-Modified Hyaluronic Acid Compounds

**DOI:** 10.3390/polym16010138

**Published:** 2023-12-31

**Authors:** Alice Donato, Antonino Di Stefano, Nadia Freato, Laura Bertocchi, Paola Brun

**Affiliations:** 1 Histology Unit, Department of Molecular Medicine, University of Padova, 35121 Padova, Italy; alicecristina.donato@phd.unipd.it; 2 Divisione di Pneumologia e Laboratorio di Citoimmunopatologia Dell’apparato Cardio Respiratorio, Istituti Clinici Scientifici Maugeri, IRCCS, 28010 Veruno, Italy; antonino.distefano@icsmaugeri.it; 3 GlycoCore Pharma, 35122 Padova, Italy

**Keywords:** hyaluronic acid, Hylach, inflammation, fibrosis, Galectin 3, IPF, prevention, pulmonary rehabilitation

## Abstract

Idiopathic pulmonary fibrosis (IPF) is a chronic inflammatory and fibrotic pathological condition with undefined effective therapies and a poor prognosis, partly due to the lack of specific and effective therapies. Galectin 3 (Gal-3), a pro-fibrotic ß-galactoside binding lectin, was upregulated in the early stages of the pathology, suggesting that it may be considered a marker of active fibrosis. In the present in vitro study, we use Hylach^®^, a lactose-modified hyaluronic acid able to bind Gal-3, to prevent the activation of lung myofibroblast and the consequent excessive ECM protein cell expression. Primary human pulmonary fibroblasts obtained from normal and IPF subjects activated with TGF-β were used, and changes in cell viability, fibrotic components, and pro-inflammatory mediator expression at both gene and protein levels were analyzed. Hylach compounds with a lactosylation degree of about 10% and 30% (Hylach1 and Hylach 2), administrated to TGF-β—stimulated lung fibroblast cultures, significantly downregulated α-smooth muscle actin (α-SMA) gene expression and decreased collagen type I, collagen type III, elastin, fibronectin gene and protein expression to near baseline values. This anti-fibrotic activity is accompanied by a strong anti-inflammatory effect and by a downregulation of the gene expression of Smad2 for both Hylachs in comparison to the native HA. In conclusion, the Gal-3 binding molecules Hylachs attenuated inflammation and TGF-β—induced over-expression of α-SMA and ECM protein expression by primary human lung fibroblasts, providing a new direction for the treatment of pulmonary fibrotic diseases.

## 1. Introduction

Idiopathic pulmonary fibrosis (IPF) is a progressive inflammatory and fibrotic disease that is associated with the deterioration of lung functions and the consequent reduction in quality of life [1]. IPF, whose incidence is increasing, mainly occurs in middle-aged and older people, with a medium survival time of 3–5 years from diagnosis [2,3].

Redundant inflammation and pathological remodeling processes in response to pro-fibrogenic cytokines play a central role in the pathogenesis of IPF. In particular, the early step of the fibrotic process is characterized by the involvement of the immune system and the differentiation of resident lung cells into contractile myofibroblasts that express the α-smooth muscle actin (α-SMA) [4,5]. These cells are of crucial importance in the progression of pathology since they are largely responsible for the deposition of an increased amount of extracellular matrix (ECM) proteins, such as collagens, elastin, and fibronectin [6]. Among the pro-fibrotic cytokines involved in the development of pulmonary fibrosis, transforming growth factor (TGF-β) is considered the most potent, playing a pivotal role in the differentiation of lung fibroblasts to myofibroblasts that stimulate ECM accumulation through the TGF-β1/SMAD signaling pathway [7,8]. Moreover, all stages of fibrosis are accompanied by an innate immune response, even though its role in the perpetuation of IPF is controversial in part [4]. Although pirfenidone and nintedanib, the only approved pharmacological therapies for IPF, are able to slow down disease progression, the pathology remains incurable [9]. Therefore, effective pharmacologic approaches are still highly needed for pulmonary fibrosis treatment, especially in preventing fibroblast differentiation to myofibroblast and the consequent excessive deposition of ECM proteins.

Galectin-3 (Gal-3), a 35-k-Da member of the β-galactoside binding lectins, is highly expressed in a variety of tissues of diverse fibrotic and inflammatory diseases [10,11,12,13]. Elevated concentrations of this galectin have been demonstrated in the serum and bronchoalveolar lavage fluid (BAL) from patients with stable IPF and even in the early stages of the pathology [14,15]. Other studies have demonstrated that Gal-3 enhanced ECM molecule synthesis by lung fibroblasts, suggesting that the molecule may be a marker of active fibrosis [16]. Taken together, these findings suggest a potential role for Gal-3 in the progression of IPF, and, as a consequence, this molecule may serve as a relevant therapeutic target for pulmonary fibrotic diseases, and its inhibition may improve disease outcomes [17].

Recently, we demonstrated that a lactose-modified hyaluronic acid (HA) named Hylach^®^ is able to bind Gal-3 and that the best ligands are the molecules with a percentage of lactosylation up to 40%. In particular, the compounds with a 10% and 30% percentage of lactose-derived residues (Hylach 1 and Hylach 2) attenuate macrophage-induced inflammation and MMP production in primary human bronchial fibroblast cultures, enhancing the well-known anti-inflammatory properties of HA and its capacity to affect ECM production [18].

Based on these findings, we hypothesized that Hylach could also reduce fibrosis by means of the interaction with Gal-3. In the present study, we aimed to in vitro evaluate the anti-fibrotic and anti-inflammatory effects of the Hylach compounds in control and IPF human fibroblasts stimulated with TGF-β in the presence or absence of HA or of Hylach with different percentages of lactosylation.

## 2. Materials and Methods

### 2.1. Chemicals and Compounds

**Figure 1:**
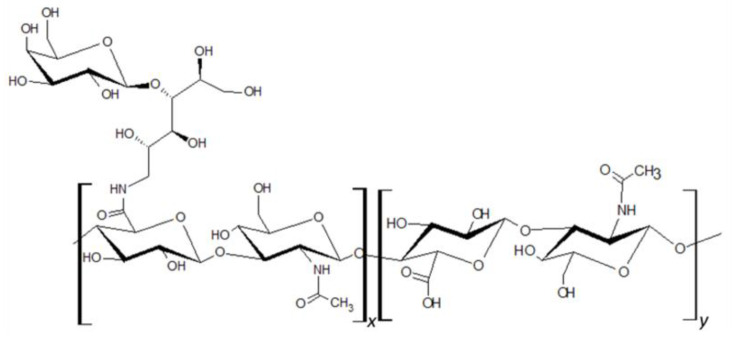
Hylach^®^ molecular structure.

HA of molecular size ranging from 80 to 150 kDa was attached to lactose-derived components (Hylach^®^ 1 and Hylach^®^ 2) with a lactosylation degree of about 10% and 30%, respectively (GlycoCore Pharma, Padova, Italy, Figure 1). Both native (82 kDa) and conjugated forms of HA were dissolved in phosphate-buffered saline before application.

### 2.2. Primary Human Pulmonary Fibroblasts and U937 Monocytes

Primary human pulmonary fibroblasts isolated from the lungs of healthy and IPF donors were purchased from Lonza (Basel, Switzerland) and cultured under standard conditions in DMEM medium (Euroclone, Pero, Italy) containing 10% fetal bovine serum (Gibco, ThermoFisher Waltham, MA, USA), 2 mM L-glutamine, 100 U penicillin and 100μg/mL streptomycin (Gibco). The human monocyte cell line U937 was obtained from Thermo Scientific (Wilmington, DE, USA) and cultured in RPMI medium (Euroclone) supplemented with 10% fetal bovine serum (Gibco) and 1% antibiotics. All cells were cultivated at 37 °C and 5% CO_2_.

### 2.3. Cell Viability Assay in the Presence of Hylach and HA Molecules

To examine the impact of Hylach 1, Hylach 2, and native HA on the viability of human pulmonary fibroblasts, cells were cultured at a density of 1200 in 96-well plates and allowed to grow under standard culture conditions for 24 h, 3 days, and 6 days with or without the presence of 0.5 mg/mL of Hylach compounds and of the native HA. Afterward, cell viability was assessed using the MTT assay (3-4,5-dimethylthiazol-2-yl-2,5-diphenyl tetrazolium bromide), following a modified Denizot method [19]. Absorbance values were determined using the Infinite F200 microplate reader (TECAN, Milan, Italy). The experiments were carried out in triplicate for each experimental condition.

### 2.4. TGF-β Stimulation of Primary Human Pulmonary Fibroblasts

Human pulmonary fibroblasts from the lungs of healthy and IPF subjects were cultured in uncoated culture flasks in standard conditions, kept in serum-free DMEM medium for 24 h, and then treated for an additional 24 h with 5 ng/mL of TGF-β (Biotechne, Minneapolis, MN, USA). The medium was subsequently removed, and the compounds derived from HA were added for 4, 10, and 24 h. Cell differentiation was evaluated through the analysis of the gene expression of α-SMA, collagen type I, collagen type III, elastin, and fibronectin.

The effects of the HA and Hylach compounds with different degrees of lactosylation on the TGF-β stimulated control and IPF human fibroblast cultures were assessed at 4, 10, and 24 h through the analysis of α-SMA, SMAD-2, collagen type I, collagen type III, elastin, fibronectin, and TGF-β expression at both gene and protein level using, respectively, qPCR and ELISA analyses.

### 2.5. Activated U937 Monocytes Conditioned Medium

U937 monocytes were differentiated in adherent macrophages with 50 ng/mL phorbol 12-myristate 13-acetate (PMA, Sigma-Aldrich, St. Louis, MO, USA) for 48 h and subsequently treated with 1 μg/mL of lipopolysaccharide (LPS, Sigma) for 1 h. Afterward, cells were cultured in complete RPMI for 24 h, and the conditioned medium (CM) was harvested, centrifuged, and filtered before human pulmonary fibroblast treatments. The differentiation of monocytes into macrophages was confirmed both under the inverted phase-contrast microscope and by assessing the CD-68 gene expression, as previously reported [20].

Lung fibroblasts from both healthy and IPF donors were treated with the CM for 24 h. Subsequently, the medium was removed and replaced with fresh medium containing HA, Hylach 1, and 2, or left untreated. The HA and Hylachs effects on human pulmonary fibroblast culture were assessed at 4 and 10 h after treatment, analyzing the expression of the pro-inflammatory molecules IL-1β, TNF-α, Gal-3, and TGF-β using qPCR.

### 2.6. RNA Isolation and qPCR Analysis

Gene expressions of inflammatory and ECM and Smad molecules by primary human pulmonary fibroblasts stimulated with TGF-β or CM of activated U937 monocytes and then treated with HA and Hylach molecules were examined using qPCR.

The total RNA was isolated according to the manufacturer’s protocol using TRIzol (Life Technologies, Carlsbad, CA, USA), and the RNA quality was assessed using the Nanodrop 2000c spectrophotometer (Thermo Scientific) by measuring the absorbance at 260/280 nm. To eliminate DNA contamination, the total RNA samples underwent a 15 min treatment with DNAse I (Thermo Fisher). Subsequently, 500 ng of total RNA was reverse transcribed into cDNA using oligo-dT and Superscript II (Life Technologies, Carlsbad, CA, USA). The gene expression levels of the pro-inflammatory and ECM molecules were quantified using the Xpert fast SYBR (GRISP, Porto, Portugal) on a Rotor-Gene RG-3000A system (QIAGEN, Hilden, Germany). The normalization of target gene expression was carried out relative to the intrinsic levels of peptidylprolyl isomerase A (PPIA). The 2^∆Ct^ method was employed for assessing gene expression, with the dCt calculated as Ct PPIA—Ct target gene. Refer to Table 1 for a comprehensive list of primers utilized in the qPCR analysis.

### 2.7. Enzyme-Linked Immunosorbent Assay (ELISA)

The expression of ECM molecules by primary human pulmonary fibroblasts stimulated with TGF-β and afterward exposed to HA and Hylach compounds were confirmed at the protein level using the ELISA assay.

Supernatants from primary human pulmonary fibroblast cultures, stimulated or not with TGF-β 5 ng/mL for 24 h and then treated with HA and Hylach for 4, 10, and 24 h, were collected. Elastin, fibronectin, and TGF-β were quantified using ELISA kits from MyBioSource (San Diego, CA, USA), COL-I using the ELISA Kit from Ray Biotech (Ray Biotech, Inc., Peachtree Corners, GA, USA), and COL-III using the human ELISA kit from Nordic BioSite (Taby, Sweden) according to the suppliers’ protocols. Each experiment was conducted in triplicate.

### 2.8. Statistical Analysis

Statistical analysis was carried out using GraphPad Prism 9 (San Diego, CA, USA), which involved employing a one-way analysis of variance (ANOVA) followed by Tukey’s post hoc test for multiple comparisons. Additionally, an unpaired Student’s *t*-test was utilized. The significance threshold in statistical analysis was set at a *p*-value below 0.05.

## 3. Results

### 3.1. TGF-β Stimulation of Lung Fibroblasts Upregulates the Expression of α-SMA and ECM Molecules

The stimulation of lung fibroblasts from IPF and normal subjects with 5 ng/mL of TGF-β induced a change in cell morphology and an intensive expression of *α-SMA*, and we found that this stimulation was less pronounced in the cells derived from IPF patients compared to those from control donors (*p* < 0.05 vs. *p* < 0.001).

**Figure 2:**
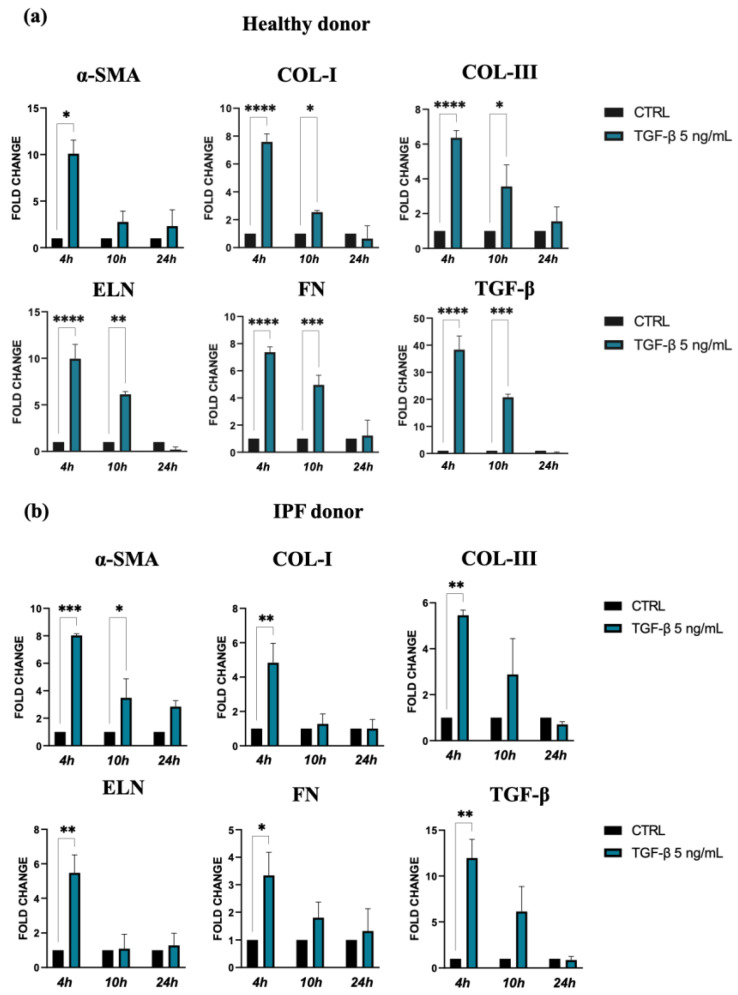
Expression of α-SMA and ECM molecules by lung fibroblast from healthy (**a**) and IPF donors (**b**) cultures exposed to 5 ng/mL TGF-β for 24 h. Quantification of RNA transcript levels was carried out using qPCR analyses. The data represent the mean ± standard error (SE) of results obtained from three independent experiments. Statistical differences were assessed using an unpaired Student’s *t*-test, and significance levels were indicated as follows: * *p* < 0.05, ** *p* < 0.01, *** *p* < 0.001, and **** *p* < 0.0001.

TGF-β and the consequent cell activation also induce a strong upregulation of gene expression for ECM molecules, such as collagen I, collagen III, elastin, and fibronectin, in both types of lung fibroblasts stimulated by TGF-β. Indeed, this effect was more marked for cells isolated from healthy donors in comparison with those from IPF patients, especially at 4 h after treatment (Figure 2a,b). Furthermore, we observed a positive feedback loop with TGF-β, as TGF-β expression was also increased in response to TGF-β treatment in both control and the IPF fibroblasts.

Taken together, the results of these experiments confirmed that TGF-β promotes in vitro differentiation of lung fibroblasts into myofibroblasts, the major source of ECM molecules during pulmonary fibrogenesis.

### 3.2. HA and Hylach at Different Percentages of Lactosylation Do Not Affect the Viability of Lung Fibroblasts

**Figure 3:**
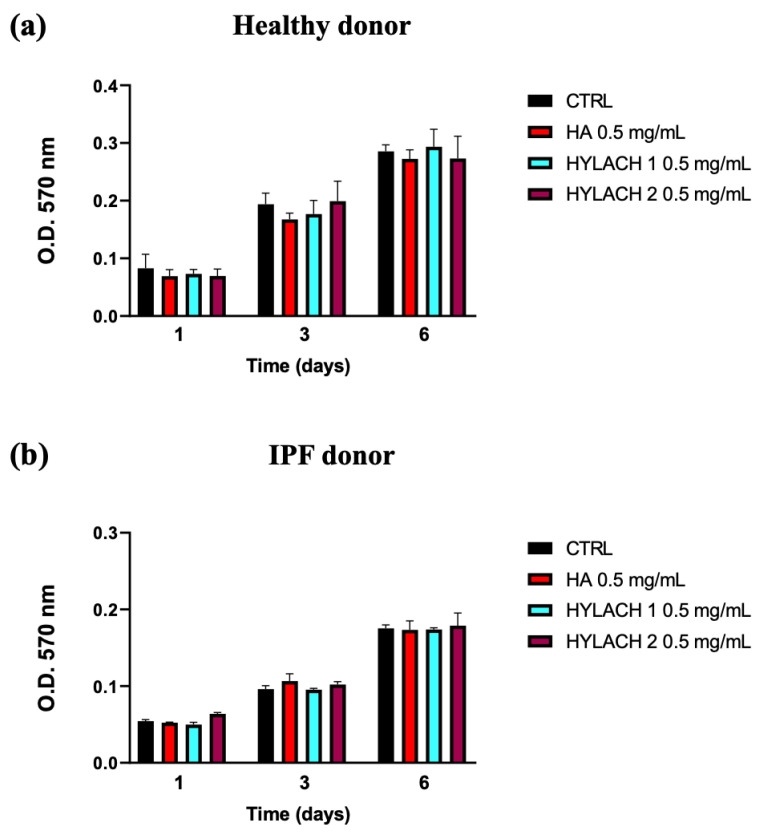
Time-dependent impact of HA and Hylach on human lung fibroblast viability. 1200 cells from both healthy (**a**) and IPF (**b**) subjects were seeded in 96-well culture plates and treated with 0.5 mg/mL HA, Hylach 1, and Hylach 2 for 1, 3, and 6 days. Cell viability was measured using an MTT assay. Data are presented as the means ± standard error (SE) from three independent experiments. Statistical variances were assessed utilizing the unpaired Student’s *t*-test. The percentage of lactose derivative residues is as follows: Hylach 1 = 10%, Hylach 2 = 30%.

Hylach 1, Hylach 2, and the native HA, administered to human primary lung control and IPF human fibroblasts cultured in standard conditions, did not significantly affect their viability after 1, 3, and 6 days of treatment (Figure 3), confirming that the HA and Hylach molecules did not exert alterations on lung fibroblasts proliferation, at the concentration of 0.5 mg/mL.

### 3.3. Hylach 1 and Hylach 2 Inhibits the Over-Expression of α-SMA and ECM Molecules in TGF-β Stimulated Lung Fibroblast Cultures

**Figure 4:**
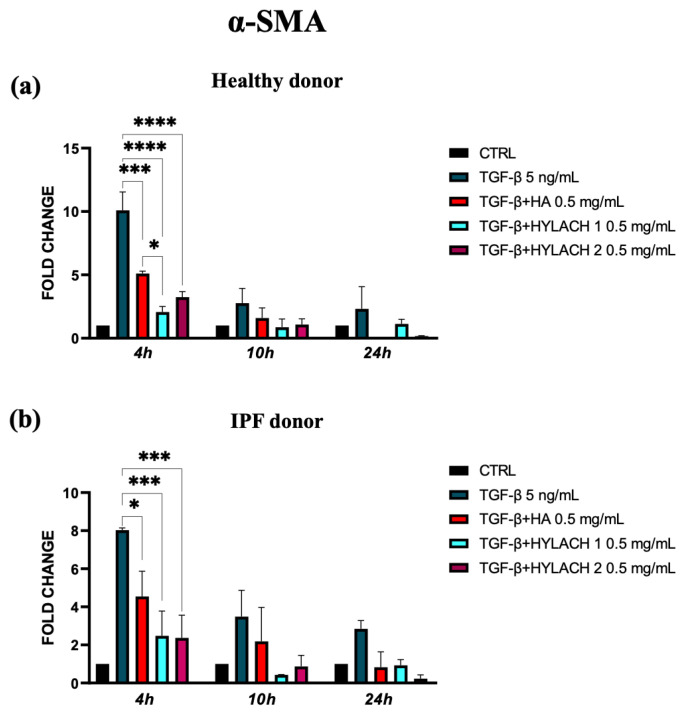
Modulation of α-Smooth Muscle Actin (α-SMA) gene expression in lung fibroblasts by Hylach and native HA. Human pulmonary fibroblasts from healthy (**a**) and IPF 8 (**b**) donors were exposed to TGF-β (5 ng/mL) for 24 h and subsequently cultured in the presence or absence of HA, Hylach 1, and Hylach 2. qPCR was employed to measure the mRNA level for α-SMA at 4, 10, and 24 h post-treatment. Statistical significance was determined via a one-way ANOVA test with multiple comparisons vs. untreated cells (* *p* < 0.05, *** *p* < 0.001, and **** *p* < 0.0001). The results represent the mean ± standard error (SE) of three independent experiments. *Percentage of lactose derivative residues*: Hylach 1 = 10%, Hylach 2 = 30%.

To explore whether the native HA and Hylach with different numbers of lactose-derived residues played a role in the downregulation of α-SMA and ECM molecule expression, we treated TGF-β stimulated lung fibroblasts with HA, Hylach 1, or Hylach 2, for 4–10 and 24 h. The qPCR analysis showed that the myofibroblast activation in response to TGF-β was significantly reduced in the presence of Hylach and HA since the α-SMA mRNA level of control and IPF human fibroblasts were downregulated (Figure 4a, *p* < 0.001 and *p* < 0.0001). Notably, the reduction is more pronounced in the presence of Hylach 1 than of HA at 4 h post-treatment for both cell cultures (*p*< 0.0001 and *p* < 0.001 vs. *p* < 0.05).

**Figure 5:**
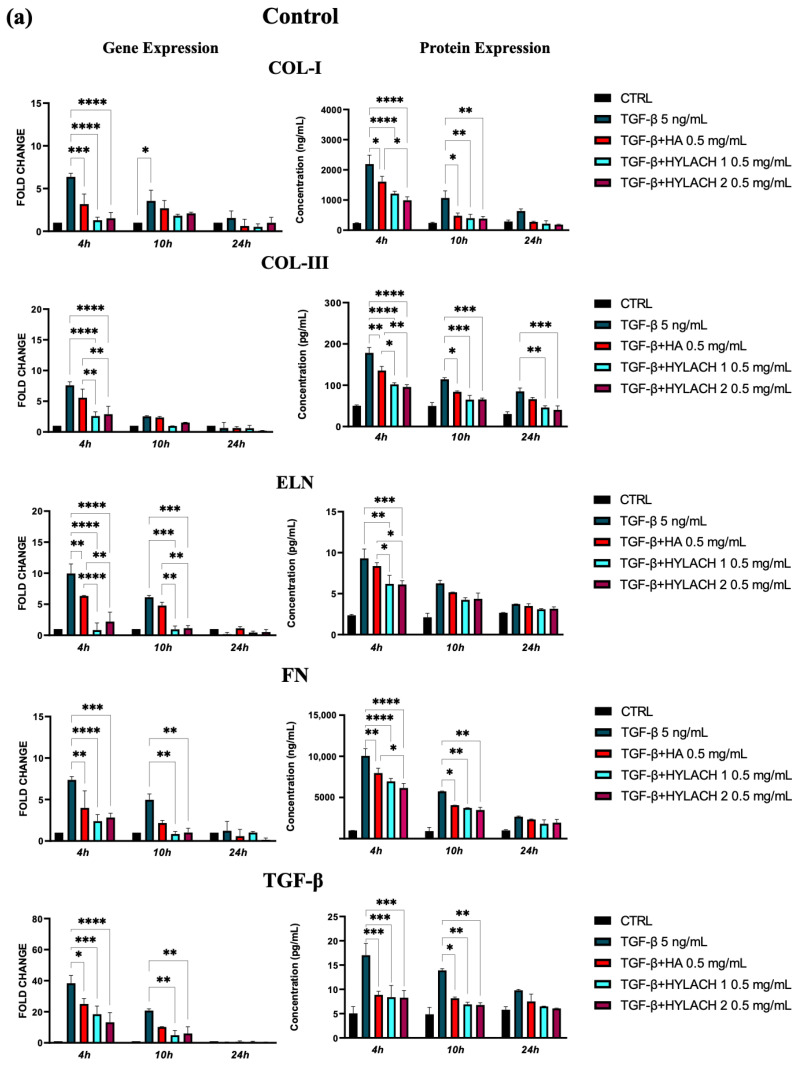

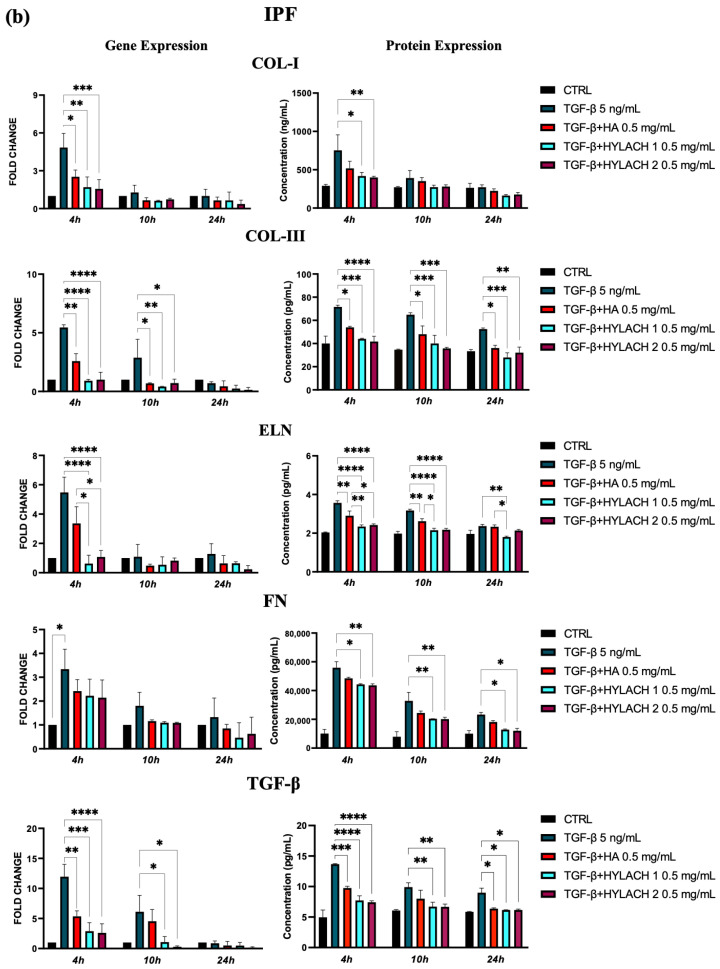
ECM protein expression by TGF-β stimulated fibroblasts treated with HA and Hylach. Human pulmonary fibroblasts from control (**a**) and IPF (**b**) donors were exposed to TGF-β (5 ng/mL) for 24 h and then cultured in the presence or absence of HA, Hylach 1 or Hylach 2. mRNA levels for collagen I, collagen III, elastin, fibronectin, and TGF-β were analyzed using qPCR at 4, 10, and 24 h post-treatment. Statistical significance was determined via a one-way ANOVA test with multiple comparisons vs. untreated cells (* *p* < 0.05, ** *p* < 0.01, *** *p* < 0.001, and **** *p* < 0.0001). The results represent the mean ± standard error (SE) of three independent experiments. *Percentage of lactose derivative residues*: Hylach 1 = 10%, Hylach 2 = 30%.

Moreover, markedly lower ECM molecule expression in HA and Hylach-treated TGF-β stimulated cells analysis was found, as judged by the qPCR and confirmed using ELISA (Figure 5). Hylach and HA significantly reduced the gene and protein expression of collagen I, collagen III, elastin, fibronectin, and TGF-β in human pulmonary fibroblasts obtained from a healthy donor, particularly at 4 h post-treatment, with the effect of Hylach being stronger than that of HA, especially where the expression of collagen I and elastin was concerned (*p* < 0.05). Furthermore, only Hylach significantly downregulates the gene and protein expression of collagen III. It is noteworthy that the anti-fibrotic effect of Hylach 2 is more potent than Hylach 1, especially where the protein expression of collagen I, collagen III, fibronectin, and TGF-β is concerned. The anti-fibrotic effect of Hylach persists even at 10 h post-treatment for collagen I, elastin, fibronectin, and TGF-β and extends to 24 h post-treatment for collagen type III. Regarding pulmonary fibroblasts from patients with idiopathic pulmonary fibrosis, both Hylach and HA significantly reduced the gene and protein expression of collagen III, elastin, and TGF-β, with the effect of Hylach being more pronounced than that of HA (*p* < 0.0001 vs. *p* < 0.01). Moreover, only Hylach is capable of downregulating both the gene and protein expression of Col-I and FN, with the anti-fibrotic effect of Hylach 2 being more effective than that of Hylach 1. Furthermore, the anti-fibrotic effect of HA and Hylach extends to 10 h and 24 h post-treatment for col-III, ELN, FN, and TGF-β. In summary, Figure 5 shows that the increase in collagen I, collagen III, and fibronectin was nearly abrogated in the lung fibroblast culture from healthy subjects (Figure 5) and slightly, but also significantly reduced in cells isolated from IPF patients (Figure 5b) in the presence of both Hylach molecules. We compared the results obtained from cells derived from healthy and IPF patients in Table 2, and we observed the most significant differences in gene and protein expression at 4 h post-treatment.

### 3.4. Hylach 1 and Hylach 2 Exert an Anti-Inflammatory Effect in Primary Human Lung Fibroblasts Cultures Exposed to CM of Activated Human Macrophages

Stimulated U937 human monocytes generate various inflammatory molecules released into the culture medium (CM), mimicking a macrophage inflammatory event, as demonstrated by previous studies [21].

**Figure 6:**
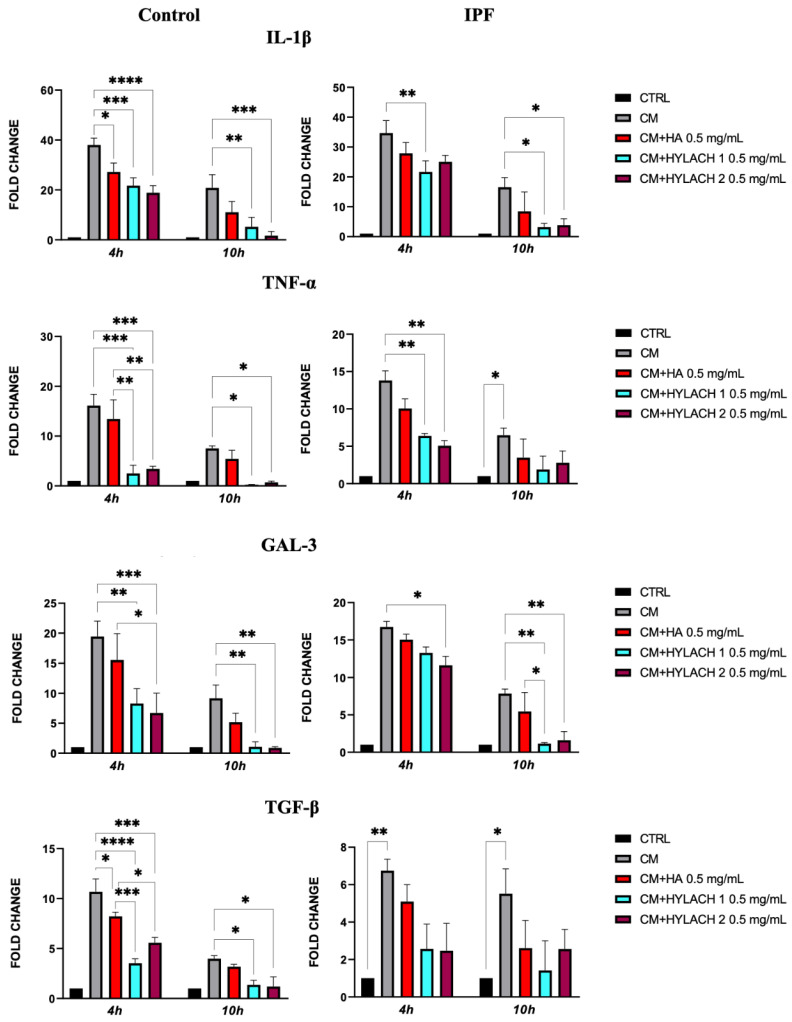
Pro-inflammatory molecule gene expression in primary lung fibroblasts following exposure to U937 CM in the presence or absence of Hylach compounds and native HA. Primary human lung fibroblasts isolated from control and IPF donors were cultivated in the presence or absence of 0.5 mg/mL HA, Hylach 1, and Hylach 2 compounds after a 24 h exposure to activated U937 CM. At 4 and 10 h after the treatment, the gene expression of IL-1β, TNF-α, TGF-β, and Gal-3 was analyzed using qPCR. Statistical significance was determined via a one-way ANOVA test with multiple comparisons vs. untreated cells (* *p* < 0.05, ** *p* < 0.01, *** *p* < 0.001, and **** *p*< 0.0001). The results represent the mean ± standard error (SE) of three independent experiments. Percentage of lactose derivative residues: Hylach 1 = 10%, Hylach 2 = 30%.

After exposing normal and IPF-derived lung fibroblasts to a conditioned medium (CM) for 24 h, a notable increase in the expression of pro-inflammatory molecules *IL-1β*, *TNF-α*, *Gal-3*, and *TGF-β* was observed—as shown in Figure 6, no difference was found in the response to the CM in lung fibroblasts isolated from IPF patients when compared to those from control donors. Afterward, HA, Hylach 1, and Hylach 2 compounds were administered to the inflamed cell cultures for 4 and 10 h, and qPCR analysis revealed that all the molecules decreased the cellular expression of all the pro-inflammatory molecules *IL-1β*, *TNF-α*, and *TGF-β* in cells from the control donors, whereas only Hylach compounds have this effect in cells isolated from IPF patients (Figure 6). Additionally, Hylach molecules, but not HA, significantly reduced Gal-3 in IPF-derived cells at 4 h, and this effect persisted at 10 h of culture.

### 3.5. The Effects of Hylach Compounds Are Correlated to the Downregulation of SMAD 2 Expression

**Figure 7:**
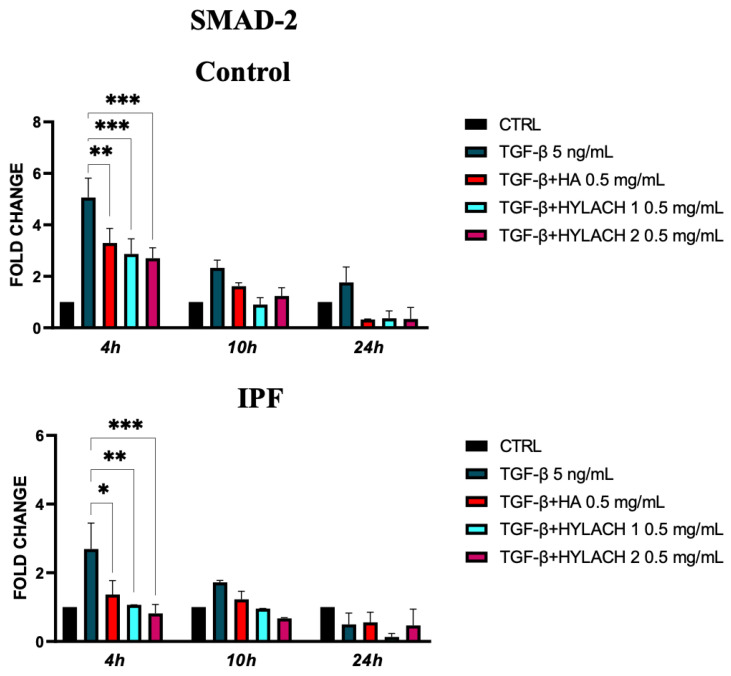
*Smad-2* expression of primary human lung fibroblasts exposed to TGF-β in the presence or absence of Hylach and HA molecules. Cell cultures were exposed for 24 h to TGF-β and then cultured in the presence or absence of 0,5 mg/mL of Hylach or HA. RNA transcript levels for *Smad-2* were assessed using qPCR at 4, 10, and 24 h post-treatment. Statistical differences were determined using the ANOVA test with multiple comparisons vs. untreated cells (* *p* < 0.05, ** *p* < 0.01 and *** *p* <0.001), and data are presented as mean ± SE obtained from three independent experiments. *Percentage of lactose derivative residues*: Hylach 1 = 10%, Hylach 2 = 30%.

We analyzed the expression of SMAD-2 molecules in TGF-β-stimulated primary human fibroblasts isolated from healthy and IPF subjects at 4, 10, and 24 h after treatment with Hylach 1, Hylach 2, and the native HA when the anti-fibrotic and anti-inflammatory effects of these molecules were more marked. We found that SMAD-2 overexpression induced by TGF-β stimulation was significantly reduced at 4 h by all the tested compounds in the control and IPF-derived fibroblast cultures (Figure 7).

## 4. Discussion

The differentiation of lung fibroblasts in myofibroblasts and the consequent upregulation of ECM protein expression is a critical process in the development of fibrosis in several fibrotic diseases and, in particular, in IPF. This chronic progressive lung disease can result in respiratory failure and even death. Therefore, strategies for contrasting pulmonary fibrosis by suppression of fibroblast differentiation and the consequent excessive ECM deposition may represent a promising therapeutic strategy. Our study demonstrated that when Hylach, a lactose-modified HA able to bind to Gal-3, was administered to TGF-β-stimulated fibroblasts derived from IPF subjects and controls, the expression of ECM components—collagen I, collagen III, elastin, and fibronectin—was dramatically reduced. We compared the activity of Hylach with that of HA, for which anti-inflammatory and protective effects are well known [22], and we found that the anti-inflammatory and anti-fibrotic effects of the lactosylated HA are higher than those exerted by the native HA.

Gal-3 is a pro-inflammatory and pro-fibrotic β-galactoside lectin molecule released following tissue injury that has been associated with the innate immune cell activation and perpetuation of chronic inflammation leading to an aberrant tissue repair, enhanced ECM molecule synthesis and, as a consequence, to fibrosis [23,24,25]. Abnormal accumulation of Gal-3 has been found in the serum and BAL from subjects with stable IPF, which was also detected in the early stages of the disease [16]. Therefore, Gal-3 acts not only as a regulator of inflammatory response in lung diseases but also as a pro-fibrotic molecule. In our study, the anti-inflammatory and anti-fibrotic effects of Hylachs are presumably due to the interaction of Gal-3 with the numerous lactose residues of the molecule. In fact, in a previous in silico study on the interaction between the Carbohydrate Recognition Domain (CRD) of Gal-3 and Hylach^®^, we found that Hylach^®^ displayed a nice fitting into the CRD active site, whereas HA molecules had only transient interactions with the target molecule. This is possibly the consequence of charges residing on the HA molecule that provide unspecific binding to Gal-3. The direct targeting of Hylach to Gal-3 significantly enhances the effects of the bare HA backbone on ECM molecules, as observed in this study [18]. We used compounds with 10% and 30% lactosylation named Hylach 1 and Hylach 2, respectively, since we found that Hylach with a percentage of lactosylation up to 10–40% are the best ligands for Gal-3. Moreover, subsequent in vitro experiments performed using human bronchial fibroblast cultures confirmed the strong anti-inflammatory effects of Hylach molecules with 10% and 30% of lactose-derived residues. In these studies, 0.5 mg/mL Hylach compounds downregulated the expression of pro-inflammatory cytokines and of Gal-3 [18]. The in vitro experiments also showed that the response of the IPF fibroblast to Hylachs was similar to that of fibroblasts obtained from healthy donors. In the present study, we chose to explore the effects of Hylach at the same concentrations since other referential dosages are not reported, and the US Food and Drug Administration advises not exceeding a 0.1% HA solution (1 mg/mL) for lung treatments.

The presented study also confirmed that TGF-β is a major inducer of lung fibroblast differentiation to matrix-secreting myofibroblasts, inducing the overexpression of α-SMA and collagen deposition and, indirectly, that Gal-3 is an essential mediator of TGF-β–induced lung fibrosis [26,27]. Moreover, we found that there was little difference in response to TGF-β in the lung fibroblasts isolated from IPF patients compared to those from control donors cultured in standard conditions, as reported by other authors [28], since in both the control and IPF fibroblasts, there was an increase in collagens, elastin, and fibronectin expression after the TGF-β challenge, followed by a significant decrease after Hylach treatments, which was higher than that exerted by the native HA. The reduction of collagen type III gene expression is higher than that of collagen type I, and this result is consistent with the fact that collagen type III is the most upregulated collagen in the first stages of fibrosis and inflammation [29]. This effect is reflected in the longer-lasting protein expression reduction in both cell cultures from healthy and IPF subjects. Furthermore, there is no difference between gene and protein expression of fibronectin in cells isolated from control subjects, while the reduction of the protein expression induced by Hylach is more relevant in the culture of cells obtained from IPF donors. We can speculate that reducing Gal-3 at the cell surface by Hylach^®^ may have an effect on fibronectin deposition since it was recently demonstrated that fibronectin provides interactions with Gal-3 [30].

Although the role of inflammation in the progression of IPF is in part controversial since some anti-inflammatory therapies have failed [31,32] to contrast the disease progression, it is well demonstrated that in fibrotic lungs, the population of lung macrophages are able to release high levels of pro-inflammatory cytokines, including IL-1β, TNF-α, and TGF-β, with the latter also having a crucial role in the induction of fibrosis [33,34]. Moreover, despite some concerns, new anti-inflammatory therapies are currently being tested, demonstrating an induction of increased pulmonary functions in patients after treatment [35]. In our study, we used the CM of activated U937 monocytes to induce in vitro inflammation in primary human lung fibroblasts, as we have previously demonstrated that this stimulus induced an increase in both pro-inflammatory molecule production and galectins [20]. With the present study, we found that when the Gal-3 binding molecules Hylach were administered to the control and IPF human fibroblasts who had earlier been exposed to the CM of activated U937 monocyte, the expressions of the pro-inflammatory cytokines IL-1β, TNF-alpha, TGF-β and Gal-3 were strongly reduced. We found that the cells isolated from IPF donors respond less to the CM stimulation in comparison to cells from healthy donors. These results are consistent with recent findings of other authors that demonstrated that IPF-derived fibroblasts exhibit reduced inflammatory responses in comparison, as reported recently by other authors who demonstrated that these alterations are due to multiple mechanisms [36]. Moreover, our study showed the Hylach 1 and Hylach 2 downregulation of Smad2 expression, supporting the hypothesis that Hylach compounds inhibit the TGF-β/SMAD signaling pathway, the main recognized pro-fibrogenic pathway in IPF. As was previously reported, activated TGF-β receptors stimulate the phosphorylation of SMAD2/3, which then translocates into the nucleus to mediate the gene expression of ECM molecules and, in particular, of collagens [8,31,37]. Our findings show that Hylachs not only reduce the pro-fibrotic effect of TGF-β but also downregulate the expression of TGF-β in an in vitro model of inflammation, strengthening their role in inhibiting the pro-fibrogenic processes. However, the therapeutic treatments that lead to a TGF-β reduction might be finely adjusted, particularly in following in vivo studies, since homeostatic activity has been reported for this and other molecules belonging to its family, to avoid the possible negative effects on cell repair mechanisms in the lung [38,39].

The present study confirmed that macrophage inflammation stimulates myofibroblast differentiation and that Gal-3 binding molecules Hylach 1 and Hylach 2 strongly downregulate the expression of pro-inflammatory molecules and the excessive pro-fibrotic ECM synthesis. Moreover, we can speculate that the lactosylated molecules exert a strong reduction of ECM molecule expression, inactivating TGF-β/SMAD signaling in the lung fibroblasts. In conclusion, taken together, these results have highlighted the role of Gal-3 in sustaining inflammation and fibrosis and may be considered as an initial step for the development of new therapeutic treatments that have to be confirmed by further in vitro and in vivo investigations.

## Figures and Tables

**Table 1:** Genes explored and primers employed.

Gene (Accession Number)	Name	Primer Sequences
*IL-1β* (NM_000576.3)	*Interleukin 1 beta*	Fw 5′-GAATCTCCGACCACCACTACAG-3′ Rv 5′-TGATCGTACAGGTGCATCGTG-3′
*LSGALS3* (NM_002306.4)	*Galectin 3*	Fw 5′-CTGCTGGGGCACTGATTGT-3′ Rv 5′-TGTTTGCATTGGGCTTCACC-3′
*TNF-α* (NM_000594.3)	*TNF- alpha*	Fw 5′-AAGCCTGTAGCCCATGTTGT-3′ Rv 5′-GGACCTGGGAGTAGATGAGGT-3′
*PPIA* (NM_021130.5)	*Peptidylprolyl Isomerase A*	Fw 5′-GGGCTTTAGGCTGTAGGTCAA-3′ Rv 5′-AACCAAAGCTAGGGAGAGGC-3′
*COL-I* (NM_000088.4)	*Collagen I*	Fw 5′-TGGAGCAAGAGGCGAGA-3′ Rv 5′-ACCAGCATCACCCTTAGCAC-3′
*COL-III* (NM_000090.4)	*Collagen III*	Fw 5′-CGGGTGAGAAAGGTGAAGGAG-3′ Rv 5′-AGGAGGACCAGGAAGACCA-3′
*ELN* (NM_000501.4)	*Elastin*	Fw 5′-CAGCTAAATACGGTGCTGCTG-3′ Rv 5′-AATCCGAAGCCAGGTCTTG-3′
*FN* (NM_001306129.2)	*Fibronectin*	Fw 5′-TCAGCTTCCTGGCACTRCTG-3′ Rv 5′-TCTTGTCCTACATTCGGCGG-3′
*TGF-β* (NM_000660.7)	*Transforming Growth Factor-β*	Fw 5′-CGACTCGCCAGAGTGGTTAT-3′ Rv 5′-AGTGAACCCGTTGATGTCCA-3′
*α-SMA* (NM_001141945.3)	*Alpha Smooth Muscle Actin*	Fw 5′-ACTGAGCGTGGCTATTCCTCCGTT-3′ Rv 5′-GCAGTGGCCATCTCATTTTCA-3′
*SMAD-2* (NM_001003652.4)	*Mothers against decapentaplegic homolog 2*	Fw 5′-TTTGCTGCTCTTCTGGCTCA-3′ Rv 5′-CCTTCGGTATTCTGCTCCCC-3′

Fw = forward; Rv = reverse.

**Table 2:** Comparison of ECM molecule expression by TGF-β stimulated fibroblasts derived from healthy and IPF patients treated with Hylach 1, Hylach 2, or HA. The arrows in the table signify the reduction in expression induced by the molecules, with the hyphen (-) denoting statistical non-significance.

Compounds		Hylach^®^ 1			Hylach^®^ 2			HA		
	**Time**	**4h**	**10h**	**24h**	**4h**	**10h**	**24h**	**4h**	**10h**	**24h**
**Normal subjects**										
**Gene expression**	COL-I	↓↓↓↓	-	-	↓↓↓↓	-	-	↓↓↓	-	-
	COL-III	↓↓↓↓	-	-	↓↓↓↓	-	-	-	-	-
	Elastin	↓↓↓↓	↓↓	-	↓↓↓	↓↓	-	↓↓	-	-
	Fibronectin	↓↓↓↓	↓↓↓	-	↓↓↓↓	↓↓↓	-	↓↓	-	-
	TGF-β	↓↓↓	↓↓	-	↓↓↓↓	↓↓	-	↓	-	-
	α-SMA	↓↓↓↓	-	-	↓↓↓↓	-	-	↓↓↓	-	-
	SMAD-2	↓↓↓	-	-	↓↓↓	-	-	↓↓	-	-
	IPF patients									
	COL-I	↓↓	-	-	↓↓↓	-	-	↓	-	-
	COL-III	↓↓↓↓	↓↓	-	↓↓↓↓	↓	-	↓↓	↓	-
	Elastin	-	-	-	-	-	-	-	-	-
	Fibronectin	↓↓↓↓	-	-	↓↓↓↓	-	-	-	-	-
	TGF-β	↓↓↓	↓	-	↓↓↓↓	↓	-	↓↓	-	-
	α-SMA	↓↓↓	-	-	↓↓↓	-	-	↓	-	-
	SMAD-2	↓↓	-	-	↓↓↓	-	-	↓	-	-
**Compounds**		**Hylach^®^ 1**			**Hylach^®^ 2**			**HA**		
	**Time**	**4h**	**10h**	**24h**	**4h**	**10h**	**24h**	**4h**	**10h**	**24h**
**Normal subjects**										
**Protein expression**	COL-I	↓↓↓↓	↓↓	-	↓↓↓↓	↓↓	-	↓	↓	-
	COL-III	↓↓↓↓	↓↓↓	↓↓	↓↓↓↓	↓↓↓	↓↓↓	↓↓	↓	-
	Elastin	↓↓	-	-	↓↓↓	-	-	-	-	-
	Fibronectin	↓↓↓↓	↓↓	-	↓↓↓↓	↓↓	-	↓↓	↓	-
	TGF-β	↓↓↓	↓↓	-	↓↓↓	↓↓	-	↓↓↓	↓	-
	**IPF patients**									
	COL-I	↓	-	-	↓↓	-	-	-	-	-
	COL-III	↓↓↓	↓↓↓	↓↓↓	↓↓↓↓	↓↓↓	↓↓	↓	↓	↓
	Elastin	↓↓↓↓	↓↓↓↓	↓↓	↓↓↓↓	↓↓↓↓	-	↓↓	↓↓	-
	Fibronectin	↓	↓↓	↓	↓↓	↓↓	↓	-	-	-
	TGF-β	↓↓↓↓	↓↓	↓	↓↓↓↓	↓↓	↓	↓↓↓	-	↓

## Data Availability

The data presented in this study are available on request from the corresponding author.
